# Equine Induced Pluripotent Stem Cells have a Reduced Tendon Differentiation Capacity Compared to Embryonic Stem Cells

**DOI:** 10.3389/fvets.2015.00055

**Published:** 2015-11-16

**Authors:** Emma P. Bavin, Olivia Smith, Arabella E. G. Baird, Lawrence C. Smith, Deborah J. Guest

**Affiliations:** ^1^Centre for Preventive Medicine, Animal Health Trust, Newmarket, UK; ^2^Département de biomédecine vétérinaire, Faculté de médecine vétérinaire, Université de Montréal, Saint-Hyacinthe, QC, Canada

**Keywords:** equine, embryonic stem cells, induced pluripotent stem cells, tendon, differentiation

## Abstract

Tendon injuries occur commonly in horses and their repair through scar tissue formation predisposes horses to a high rate of re-injury. Pluripotent stem cells may provide a cell replacement therapy to improve tendon tissue regeneration and lower the frequency of re-injury. We have previously demonstrated that equine embryonic stem cells (ESCs) differentiate into the tendon cell lineage upon injection into the damaged horse tendon and can differentiate into functional tendon cells *in vitro* to generate artificial tendons. Induced pluripotent stem cells (iPSCs) have now been derived from horses but, to date, there are no reports on their ability to differentiate into tendon cells. As iPSCs can be produced from adult cell types, they provide a more accessible source of cells than ESCs, which require the use of horse embryos. The aim of this study was to compare tendon differentiation by ESCs and iPSCs produced through two independent methods. In two-dimensional differentiation assays, the iPSCs expressed tendon-associated genes and proteins, which were enhanced by the presence of transforming growth factor-β3. However, in three-dimensional (3D) differentiation assays, the iPSCs failed to differentiate into functional tendon cells and generate artificial tendons. These results demonstrate the utility of the 3D *in vitro* tendon assay for measuring tendon differentiation and the need for more detailed studies to be performed on equine iPSCs to identify and understand their epigenetic differences from pluripotent ESCs prior to their clinical application.

## Introduction

Tendon injuries account for up to 46% of all limb injuries in racing Thoroughbreds (1) and commonly occur in other competition horses (2, 3). Injuries heal through the formation of scar tissue which is biomechanically inferior to healthy tendon and predisposes horses to a high re-injury rate of up to 67% (2). Tendon injuries are therefore the number one reason for retirement from racing (4) and have a significant welfare impact.

Mesenchymal stem cells (MSCs) are being used increasingly for the treatment of tendon injuries in horses (5). Currently, the therapeutic administration of MSCs uses autologous cells. Bone marrow must be aspirated from each horse, with the associated risks and potential complications (6) of a relatively invasive procedure, followed by 2–4 weeks for MSC culture expansion. This process precludes the treatment of acute injuries and makes it difficult to standardize. As a result, in recent years, attention has turned toward the use of allogeneic stem cells which could provide an “off the shelf” treatment. Numerous studies have demonstrated the *in vitro* (7–10) and *in vivo* (11–15) safety of allogeneic MSCs. However, we and others have previously demonstrated that MSCs have a poor survival in the injured equine tendon (15, 16) and so are likely to function through trophic effects rather than direct differentiation.

Embryonic stem cells (ESCs) have been isolated from the inner cell mass of equine blastocysts (17, 18) and following their injection into the injured equine tendon have a high survival without inducing a cell mediated immune response or undergoing uncontrolled proliferation (15). The ESCs appear to undergo tenocyte differentiation *in vivo* (19) and can differentiate *in vitro* into functional tenocytes in response to transforming growth factor-β3 (TGF-β3) and three-dimensional (3D) culture in a collagen matrix (19, 20). Equine ESCs and their spontaneously differentiated derivatives are immune privileged *in vitro* (9) and may therefore provide an allogeneic source of cells for use in regenerative therapies to aid tendon tissue repair.

More recently, equine induced pluripotent stem cells (iPSCs) have been derived by us (21) and others (22, 23) through the overexpression of pluripotency factors in differentiated cells. Like ESCs, equine iPSCs can proliferate indefinitely *in vitro*, differentiate into derivatives of all three germ layers and may also be immune privileged (24). However, unlike ESCs they do not require the destruction of an embryo for their isolation, which makes them more accessible to researchers. The potential of equine iPSCs to be used as a source of cells, such as neurons, for cell replacement therapies is therefore being investigated (25).

In this project, our aim was to determine if iPSCs generated by two independent methods, using *piggybac* transposons and retroviral vectors, responded to the same signaling mechanisms as ESCs and could differentiate into functional tendon cells with a similar efficiency.

## Materials and Methods

This study was carried out in accordance with the recommendations of Animal Health Trust Ethical Review Committee. The protocol was approved by the Animal Health Trust Ethical Review Committee (02_2012).

### ESC Culture

Three lines (i.e., derived from three different individuals) of previously characterized ESCs (9, 15, 17–20) were used in this study. ESCs were cultured on mitotically inactivated mouse embryonic fibroblasts at 37.5°C, 5% CO_2_ as previously described (19). Briefly, cells were cultured in ESC medium [Dulbecco’s modified Eagle medium (DMEM)/F12 containing 15% fetal bovine serum, 2 mM l-glutamine, 1% non-essential amino acids, 1 mM sodium pyruvate, 0.1 mM 2-mercaptoethanol (all from Invitrogen, Renfrewshire, UK), and 1000 U/ml leukemia inhibitory factor (LIF) (Sigma, Dorset, UK)]. ESCs were passaged mechanically every 5–7 days in the presence of 2 μM Thiazovivin (StemGent, Cambridge, MA, USA). ESCs were used at passage 12–24 for all tendon differentiation studies.

### iPSC Generation and Culture

Three lines of previously characterized (21, 24) iPSCs derived from equine fetal fibroblasts using *piggybac* (PB) transposons were used in this study and cultured as previously described (21). Media consisted of DMEM high glucose supplemented with 2 mM GlutaMax™, 0.1 mM non-essential amino acids, 0.1 mM 2-mercaptoethanol, 1 mM sodium pyruvate, 50 U/ml penicillin/streptomycin, 15% fetal bovine serum (all from Invitrogen), 1000 U/ml LIF (Sigma), 10 ng/ml bFGF (Peprotech, NJ, USA), 1.5 μg/ml doxycycline (Sigma), 3 μM GSK inhibitor, 0.5M MEK inhibitor, 2.5 μM TGF inhibitor, and 2 μM thiazovivin (all from StemGent).

Three lines of iPSCs were also generated from equine fibroblasts by retroviral transduction using methods as reported previously (26). Fibroblasts were isolated from skin biopsies of two adult horses at postmortem and from the limb buds from one, day 35 horse embryo. Tissue was dissected into small pieces prior to incubation in media [DMEM high glucose, supplemented with 10% fetal calf serum, 1% penicillin–streptomycin, 2 mM l-glutamine, and 1% fungizone (all from Invitrogen) and containing 1 mg/ml collagenase type I from *Clostridium histolyticum* (Sigma)] at 37°C overnight. Cells were then resuspended in normal cell culture media (as above but without fungizone) and cultured in a 10-cm plate at 37°C, 5% CO_2_ until confluent. Fibroblast cells were passaged at confluency with trypsin-EDTA (Sigma) for expansion, and stocks were frozen in media with 10% DMSO. iPSC generation was performed as described in Ref. (26). Briefly, 4 × 10^5^ packaging cells (phoenix gag-pol cells) were plated onto five 6 cm plates (in DMEM, 10% fetal calf serum, 2 mM l-glutamine) and incubated overnight at 37°C, 5% CO_2_ prior to transfection with plasmid vectors containing 3 μg of one of the following human transcription factors: pMXs.hOct 4 (Addgene 17217), pMXs.hSox2 (Addgene 17218), pMXs.hKlf4 (Addgene 17219), and pMXs.hc-myc (Addgene 17220), or with 3 μg of pMX.GFP (Cell Biolabs) to monitor efficiency of the initial transfection of the packaging cells and the subsequent viral transduction of the equine cells. The viral vectors were pseudotyped with the VSVg envelope protein by including 3 μg of pVPack-VSV-G (Stratagene, Cheshire, UK) to each transfection reaction. Transfections were carried out using lipofectamine 2000 and Opti-MEM media (both Invitrogen) according to the manufacturer’s instructions. After 24 h, the percentage of GFP positive cells was calculated in a minimum of 10 random fields to calculate the transfection efficiency of the packaging cells.

After 48 h culture supernatant containing the viral particles was filtered through a 0.45-μM filter (Nalgene). Filtered supernatant from each transfected plate was pooled, supplemented with 1 μg/ml polybrene (Sigma) and used to infect the equine skin fibroblasts which had been plated at a density of 1 × 10^4^ the day before infection. Three rounds of viral infection were carried out at 48 h intervals. The efficiency of viral transduction of the fibroblasts was determined by calculating the percentage of GFP positive cells in a minimum of 10 random fields. Four days after the last infection, 5 × 10^3^ equine cells were plated onto 10 cm plates pre-seeded with feeder cells (mitotically inactivated mouse embryonic fibroblasts). A proportion of the infected equine cells were plated directly onto gelatin-coated coverslips and fixed for immunocytochemistry after 48 h to confirm the expression of the human-reprograming factors in the infected cells.

Two days after plating the equine fibroblasts onto feeders, the media were replaced with iPSC media [DMEM/F12, supplemented with 15% FCS, 2 mM l-glutamine, 1% non-essential amino acids, 1 mM sodium pyruvate, 0.1 mM 2-mercaptoethanol (all Invitrogen), 1000 U/ml LIF (Sigma), and 10 ng/ml basic fibroblast growth factor (bFGF, Peprotech)]. iPSC media were replaced every other day until iPSC colonies began to appear (approximately 3 days after adding iPSC media). About 5–10 days after becoming apparent, when they had reached a large enough size, a pulled Pasteur pipette was used to manually pick selected colonies. These colonies were expanded in six-well plates preseeded with feeders in iPSC media, and media were replaced every other day. When the colonies had reached a sufficient size, they were mechanically passaged.

Induced pluripotent stem cells were cultured for more than 20 passages with repeated freeze/thaw cycles carried out. Freezing was done using iPSC media containing 10% DMSO. iPSCs were used at passage 3–13 for all tendon differentiation studies.

### Karyotyping

The cells were incubated for 2 h at 37°C in media containing colcemid (0.13 μg/ml, Life Technologies, CA, USA) before harvesting with trypsin. The cells were suspended in 4 ml of 0.075M KCl and incubated at 37°C for 25 min. Ten drops of fixative (3:1 methanol:acetic acid) were added, inverted to mix and incubated at room temperature for 10 min. Following centrifugation, the cells were resuspended in 4 ml of fixative and incubated at room temperature for 30 min. The cells were then centrifuged again, and the pellet resuspended in 2 ml of fixative before chromosomes were visualized using DAPI staining.

### Two-Dimensional Differentiation

Two-dimensional (2D) differentiation studies were performed as detailed in Ref. (19, 26). Briefly, pluripotent stem cells were induced to differentiate by passaging onto gelatin-coated coverslips (Sigma) without feeder cells and in media lacking LIF, bFGF, doxycycline, and inhibitors. For spontaneous differentiation, the cells were cultured for 7–14 days before being fixed and used in immunocytochemistry. For tendon differentiation, cells were cultured for 3 days to allow their attachment before the addition of 20 ng/ml TGF-β3 (PeproTech). The differentiating cells were then harvested for RNA after 1, 3, 7, or 14 days as detailed below or fixed and used in immunocytochemistry after 7 days. Differentiation (spontaneous and tendon) was performed on three lines of each pluripotent cell type.

### Immunocytochemistry

Cells were cultured on gelatin-coated (Sigma) coverslips and fixed in 3% paraformaldehyde in PBS for 20 min at room temperature prior to permeabilization for 1 h with 0.1% triton-X-100 at room temperature. They were then washed thoroughly in PBS and incubated with the primary antibodies overnight at 4°C, before detection with an appropriate fluorescently labeled secondary antibody. All antibodies were used at optimized concentrations in PBS, and appropriate negative controls were performed using secondary antibodies alone and IgG matched to the host species and specific isotype of the primary antibody. To provide positive controls for the pluripotency markers, we used equine ESCs (17). Coverslips were mounted using Vectashield Hardset mounting medium containing DAPI (4′,6-diamidino-2-phenylindole, Vector Laboratories, Cambridge, UK). Primary antibodies included: rabbit anti-alpha-fetoprotein 1:500 (Biorbyt, Cambridge, UK), mouse anti-actin 1:200 (Dako, Cambridge, UK), mouse anti-Beta-III tubulin 1:100 (Sigma), mouse anti-SSEA-4 1:100 (Chemicon, Hampshire, UK), mouse anti-SSEA-1 1:100 (Chemicon), rat anti-SSEA-3 1:100, mouse anti-TRA-1-60 1:500 and mouse anti-TRA-1-81 1:500 (both kindly provided by Prof. Peter Andrews at the University of Sheffield, UK), rabbit anti-Oct-4 1:100 (Santa Cruz Biotechnology, CA, USA), rabbit anti-Klf-4 1:50 (Abcam, Cambridge, UK), rabbit anti-c-myc 1:100 (Abcam), rabbit anti-Sox-2 1:1000 (Abcam), rabbit anti-scleraxis 1:100 (Abcam), rabbit anti-tenascin-C 1:100 (Abcam), rabbit anti-tenomodulin 1:100 (Santa Cruz), mouse anti-collagen I 1:100 (Abcam), rabbit anti-thrombospondin-4 1:100 (Santa Cruz), and rabbit anti-COMP 1:500 (kindly provided by Professor Roger Smith, Royal Veterinary College, UK). Secondary antibodies included goat anti-mouse FITC 1:200 (Abcam), goat anti-rabbit FITC 1:100 (Sigma), goat anti-rabbit alexafluor 594 1:200 (Invitrogen), goat anti-mouse alexafluor 594 1:200 (Invitrogen), and goat anti-rat Texas Red 1:200 (Sigma).

Alkaline phosphatase was detected using the Chemicon kit according to the manufacturer’s instructions.

### RNA Extraction, cDNA Synthesis, and Quantitative PCR

This was performed as described previously (20). RNA was extracted using Tri-reagent (Sigma) and treated with Ambion DNA-free (Life Technologies, Paisley, UK). cDNA was made from 1 μg of RNA using Moloney murine leukemia virus reverse transcriptase (Promega, Hampshire, UK) with oligo(dT) and random hexamers as primers (both Promega). About 2 μl aliquots of cDNA were used in quantitative PCR (qPCR). Primers were designed using primer3^1^ and mfold^2^ to obtain amplicons with a melting temperature (Tm) of 58–62°C, devoid of secondary structure at Tm 60°C and with an amplicon size of 50–150 bp. Primer sequences can be found in Table 1. qPCR was carried out using SYBR Green containing supermix (Bioline, London, UK) on a Quantica machine (Techne, Staffordshire, UK). All PCRs were performed in duplicate. PCR cycle parameters were 95°C for 10 min, followed by 40 cycles of 95°C for 15 s, 60°C for 15 s, and 72°C for 15 s. At the end of the program, a melt curve was produced by taking readings every 1°C from 65 to 95°C. 18s rRNA levels did not change between treatments (data not shown) and was used to normalize gene expression using the 2^−ΔΔCt^ method (27). A Student’s *t*-test was used to determine statistically significant fold changes in gene expression between the control and treated groups at each time point. qPCR was performed on three lines of iPSCs and ESCs.

**Table 1:** **Primer sequences for equine gene transcripts**.

Gene	Forward	Reverse
18s rRNA	CCCAGTGAGAATGCCCTCTA	TGGCTGAGCAAGGTGTTATG
Scleraxis	CCCAAACAGATCTGCACCTT	ATCCGCCTCTAACTCCGAAT
Tenascin-C	AACCCGTCCAAAGAGACCTT	GCGTGGGATGGAAGTATCAT
Tenomodulin	GTCCCTCAAGTGAAGGTGGA	CCTCGACGGCAGTAAATACAA
Collagen type-1 alpha 1	TGCGAAGACACCAAGAACTG	GACTCCTGTGGTTTGGTCGT
Cartilage oligomeric matrix protein	AGAACATCATCTGGGCCAAC	CGCTGGATCTCGTAGTCCTC
Thrombos pondin-4	GGGAAATGGGGTTACCTGTT	CGGGTAGCAGGGATGATATT

### Three-Dimensional Differentiation

For embryoid body formation, the iPSCs were passaged onto low attachment plates (Corning Life Sciences, MA, USA) in media without feeders, LIF or bFGF. Embryoid body formation was monitored for 14 days; media was changed every 3 days.

For tendon differentiation, 0.2 mm diameter minutien pins were embedded in silicone-coated 6-well plates (Sylgard 184 Silicone elastomer, Dow Corning, USA) in pairs 15 mm apart. Mechanically passaged iPSCs were isolated, and a small aliquot was dissociated with TrypLE™ Express (Invitrogen, Renfrewshire, UK) to generate representative cell counts. Colonies of iPSCs were suspended in a chilled mixture of two parts iPSC medium (without LIF, bFGF) and eight parts PureCol (Bovine collagen type I, Advanced Biomatrix, USA) (with the pH adjusted to 7.2–7.6) at 4 × 10^5^ cells/ml. About 200 μl of collagen-iPSC suspension was pipetted between each pair of minutien pins. The six-well plates were then incubated at 37.5°C for 60–90 min to allow the constructs to set. After setting, 3 ml of iPSC medium (without LIF/FGF) was added to each well, with or without 20 ng/ml (19, 20) TGF-β3. Constructs were cultured for up to 28 days and during this time the media was changed every 3–4 days.

### Contraction Analysis

The 3D constructs were photographed daily and the images analyzed using ImageJ software (National Institutes of Health, USA). Individual images were calibrated using the well diameter. For each construct, three measurements were made across the width and averaged. Contraction of the constructs was calculated as a percentage of the Day 0 value. A Student’s *t*-test was used to determine statistically significant differences in contraction between ESCs and iPSCs. For contraction analysis, repeats were performed on three independent lines of ESCs and iPSCs with a minimum of two technical repeats carried out for each cell line. For each technical repeat, a minimum of three constructs were measured at each time point for each condition.

### Cell Survival Assay

Constructs were harvested and digested in 1 ml medium with 1 mg/ml type I collagenase produced by *C. histolyticum* (Sigma) for 1–2 h at 37.5°C. The digest was pelleted by centrifugation and resuspended in 1 ml TrypLE™ Express (Invitrogen, Renfrewshire, UK) and also kept at 37.5°C in order to fully dissociate the cells. Cell counts were performed on a hemocytometer, and results are displayed as a percentage of the seeded cell number on Day 0. A Student’s *t*-test was used to determine statistically significant differences in cell survival. Cell survival was performed using three independent cell lines of ESCs or iPSCs. For each line, a minimum of two constructs was used to calculate the cell survival at each time point for each condition.

### Histology on 3D Constructs

As described previously (20), constructs containing ESCs were embedded in OCT compound (VWR, PA, USA) and snap frozen in liquid nitrogen cooled-isopentane. A cryostat was used to cut longitudinal sections of 11 μm thickness. The sections were fixed in 100% acetone for 10 min and stored at −20°C until used. Histological staining was carried out using hematoxylin and eosin (H&E) to view the distribution and morphology of the cells.

## Results

### Generation and Characterization of Equine iPSCs From Adult Fibroblasts Using Retroviral Transduction

**Figure 1:**
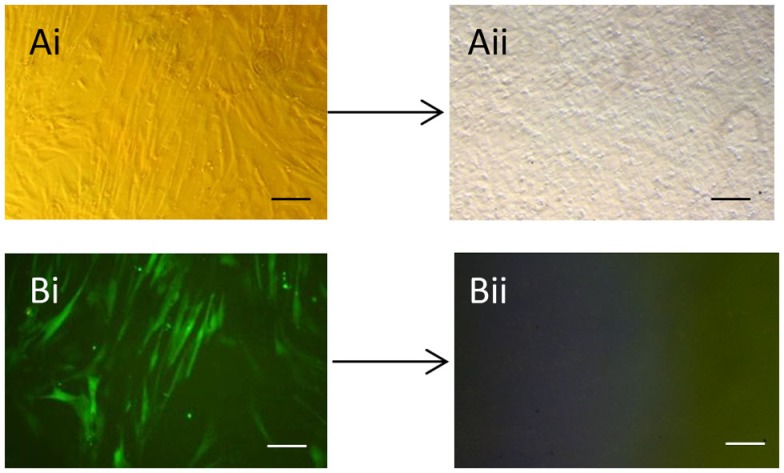
**Generation of equine iPSCs using retroviral overexpression of pluripotency genes**. **(Ai)** Equine skin fibroblasts express GFP in a high percentage of cells after retroviral transduction **(Bi)**. **(Aii)** Shows an iPSC colony which has silenced the exogenous retroviral genes and lost GFP expression **(Bii)**. Scale bar = 40 μm.

As expected, the efficiency of transfection of the packaging cells was high with >90% of the cells expressing GFP (data not shown). We found that repeated rounds of retroviral infection of the equine cells led to increased levels of GFP, with a maximum of approximately 80% being reached after three rounds of infection (Figure S1 in Supplementary Material). After three rounds of infection, a proportion of cells were used in immunocytochemistry which confirmed the expression of all four pluripotency reprograming factors in the equine cells (Figure S2 in Supplementary Material). Approximately 5 days after plating the infected equine cells onto feeders, it could be seen that a small proportion of the cells had a distinct change in their cellular morphology; going from a fibroblastic shape to forming colonies of small round cells (Figures 1Ai,Aii). Over the following days in culture the colonies increased in size and number and were selected and mechanically picked from the fibroblast culture when they were clearly visible by eye. The colonies themselves were flat, resembling human iPSCs and equine ESCs, and contained cells which had a high nuclear to cytoplasmic ratio. The equine iPSCs could be expanded for over 20 passages using mechanical passaging. During this time, the iPSCs showed no loss of their proliferative potential, with the time between passages remaining constant (5–7 days) as the passage number increased. The iPSCs could also be frozen and thawed with no change in their characteristics. From passage five, in media containing LIF and bFGF, the retroviral-derived iPSCs began to silence the expression of the integrated reprograming factors. This was visualized by a lack of GFP fluorescence in the iPSC colonies (Figures 1Bi,Bii).

**Figure 2:**
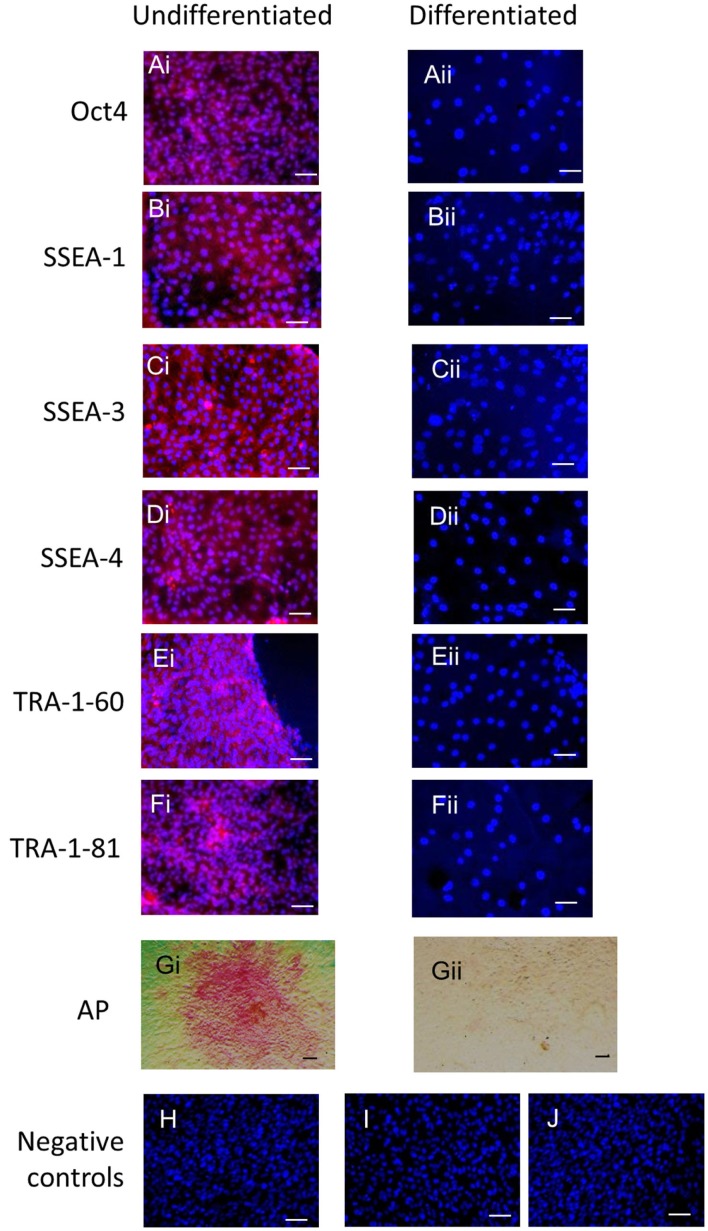
**Undifferentiated iPSCs express pluripotency markers (Ai–Gi) which are lost upon spontaneous differentiation (Aii–Gii)**. Negative controls on the undifferentiated cells for the secondary antibodies are shown in **(H)** anti-mouse, **(I)** anti-rabbit, and **(J)** anti-rat. Scale bar = 40 μm.

**Figure 3:**
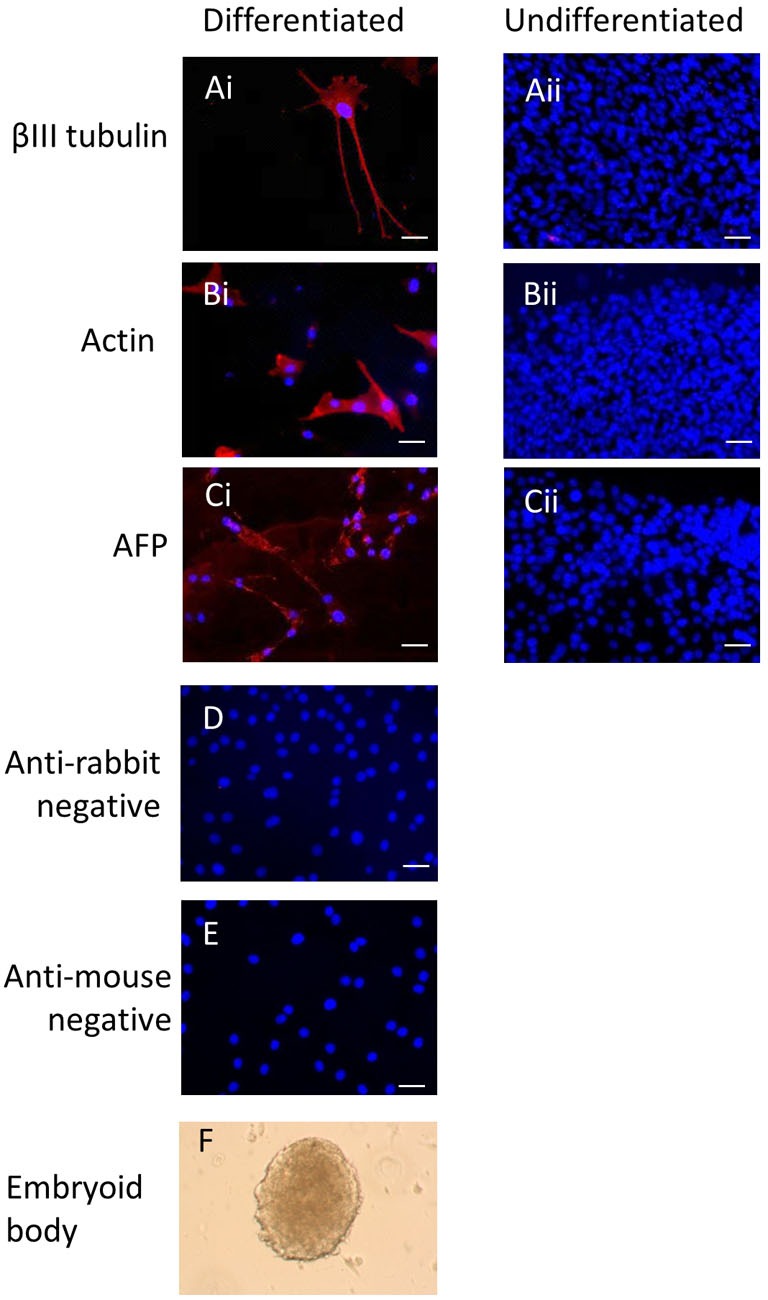
**Induced pluripotent stem cells can undergo differentiation *in vitro***. After 7 days of spontaneous *in vitro* 2D differentiation, immunocytochemistry demonstrates positive staining for βIII tubulin, actin, and alpha-fetoprotein **(Ai–Ci)**. These proteins are not produced by the undifferentiated iPSCs **(Aii–Cii)**. Negative controls on the differentiated cells are shown in **(D,E)**. Scale bar = 40 μm. The iPSCs can also form cystic embryoid bodies in suspension culture over a period of 14 days **(F)**.

**Figure 4:**
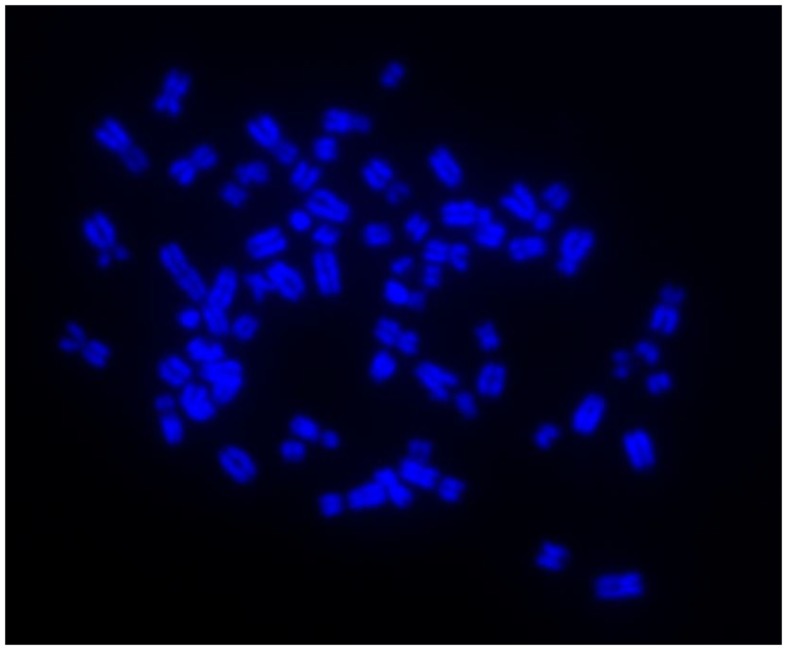
**Induced pluripotent stem cells have a normal karyotype of *n* = 64 as shown by DAPI staining of metaphase chromosome spreads**.

Immunocytochemistry on later passage cells (>5) continued to demonstrate positive staining of the undifferentiated iPSCs for pluripotency markers Oct 4, TRA-1-60, TRA-1-81, SSEA-4, SSEA-3, and SSEA-1, and staining for alkaline phosphatase activity was detected (Figures 2Ai–Gi). Together, this suggests that while the exogenous reprograming factors are silenced, the endogenous pluripotency factors remain expressed. After spontaneous *in vitro* differentiation of the iPSCs for 7 days, the expression of all pluripotency markers was lost (Figures 2Aii–Gii). Furthermore, after this time, immunocytochemistry-detected differentiation into cells derived from all three germ layers: α-fetoprotein positive fetal liver cells (endoderm), actin positive muscle cells (mesoderm), and β-III tubulin positive neuronal cells (ectoderm) (Figures 3Ai–Ci) which were not produced by the undifferentiated cells (Figures 3Aii–Cii) or the negative controls (Figures 3D, E). When plated onto low attachment plates in media without LIF or bFGF the iPSCs able to form cystic embryoid bodies over a period of 14 days (Figure 3F). Karyotype analysis of the iPSCs confirmed that they had a normal chromosome number (*n* = 64, Figure 4).

### iPSCs Generated by Different Methods Have a Similar Response to TGF-β3 in 2D Culture, But This Appears to Be Delayed Compared to ESCs

**Figure 5:**
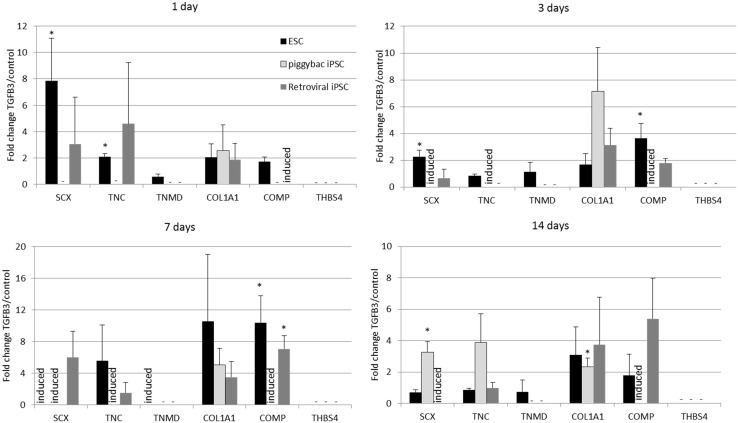
**Induced pluripotent stem cells generated by two independent methods show a similar expression profile of tendon-associated genes following 2D differentiation in the presence of TGF-β3**. But they are delayed in comparison to ESCs. Expression is shown as the fold change in TGF-β3-treated cells compared to cells differentiating in the absence of TGF-β3. *p < 0.05; −, expression not detected; induced, expression only detected in TGF-β3-treated cells.

Undifferentiated iPSCs derived by either method do not express any tendon-associated genes or proteins (data not shown) but exhibit a similar response in their tendon gene expression profiles upon the induction of differentiation in the absence and presence of TGF-β3 (Figure 5). Retroviral iPSCs, piggybac iPSCs, and ESCs all fail to demonstrate detectable expression of *THBS4* at any time point during the differentiation process in either control or TGF-β3 treated cells. In contrast, *COL1A1* is expressed in control and TGF-β3 treated cells in all cell types at all time points after the induction of differentiation. However, for all other tendon genes, there appears to be some differences between ESCs and iPSCs. The ESCs express *TNMD* throughout their differentiation, whereas neither type of iPSCs expressed detectable *TNMD* at any time during their differentiation. *TNC* is expressed in both control and TGF-β3-treated ESCs at all time points studied, whereas its detection is more variable in the iPSCs, only appearing in both control and TGF-β3-treated cells in both retroviral and *piggybac* cells after 14 days. *COMP* expression is also robust in control and TGF-β3 ESCs at all time points but is only detected in TGF-β3-treated *piggybac* iPSCs not control cells at any time point. However, after 14 days in the presence of TGF-β3, the relative expression levels of the expressed genes are not significantly different between the ESCs and iPSCs (data not shown).

**Figure 6:**
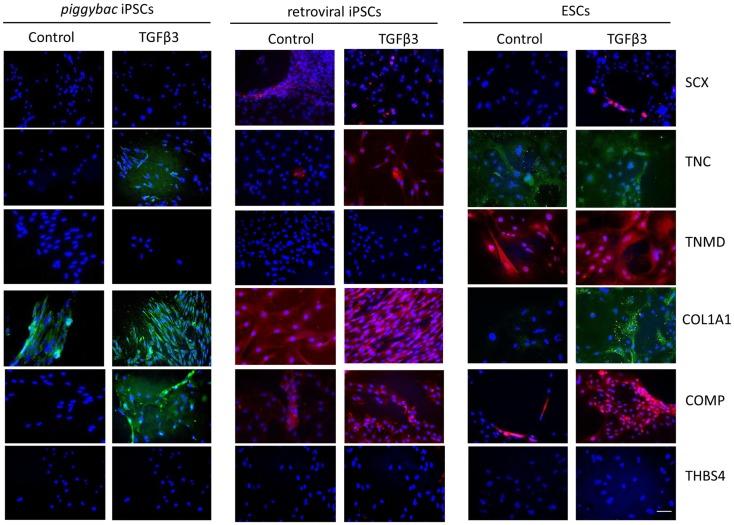
**Immunocytochemistry for protein expression in the iPSCs generated through *piggybac* transposons or retroviral vectors demonstrates largely similar profiles after 7 days of culture in the presence of TGF-β3 and supports the gene expression data**. Unlike the ESCs, neither type of iPSCs expresses TNMD protein. Scale bar = 40 μm.

Immunocytochemistry performed after 7 days of differentiation in the presence or absence of TGF-β3 (Figure 6) supports the gene expression data and demonstrates that iPSCs generated by either method show a similar protein expression profile after 7 days of culture in the presence of TGF-β3. Unlike the ESCs, the iPSCs do not express any detectable levels of TNMD. TNC and COMP are more strongly detected in the TGF-β3 group in both iPSC types, but COL1A1 is strongly detected in control and TGF-β3 in both iPSC types.

### ESCs Generate Artificial Tendons in 3D Culture More Efficiently Than iPSCs

**Figure 7:**
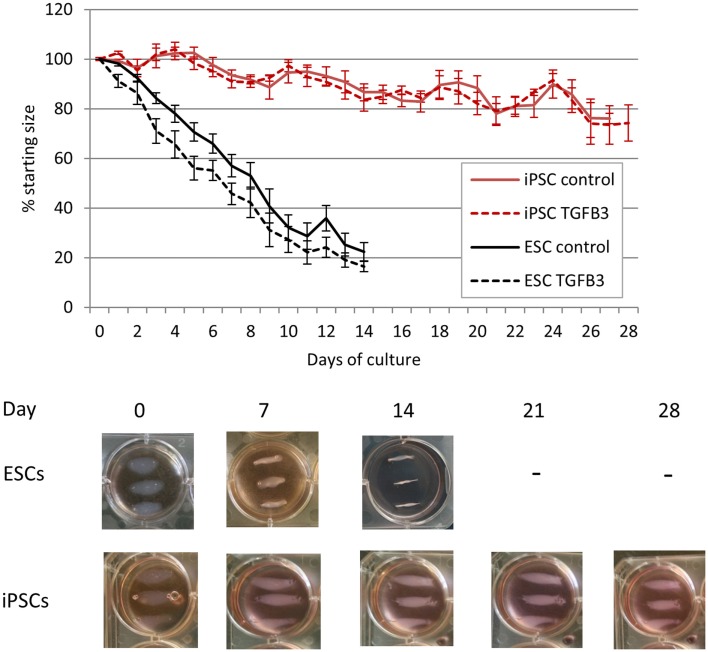
**Induced pluripotent stem cells are less efficient at remodeling a 3D collagen gel than ESCs**. ESC-seeded constructs reach their maximum contraction after 14 days of culture, whereas iPSC-seeded gels display limited contraction even after 28 days of culture.

As the iPSCs derived from retroviral transduction of adult fibroblasts behaved similar to iPSCs derived from fetal fibroblasts by transposons, only the retrovirally generated iPSCs were used in 3D cultures. ESCs cultured in a 3D collagen gel form artificial tendons that are indistinguishable from those generated by adult tenocytes over a period of 14 days (20). iPSCs cultured in 3D collagen gels undergo significantly slower re-organization of the collagen matrix than ESCs (Figure 7). Whereas ESCs have contracted the matrix to the maximum (approximately 20% of its starting size) after 14 days, iPSCs fail to reach maximum contraction even after 28 days of culture. ESCs cultured in 3D in the presence of TGF-β3 have an enhanced rate of early tendon differentiation (20), but TGF-β3 has no significant effect on the contraction rate of 3D gels containing iPSCs.

**Figure 8:**
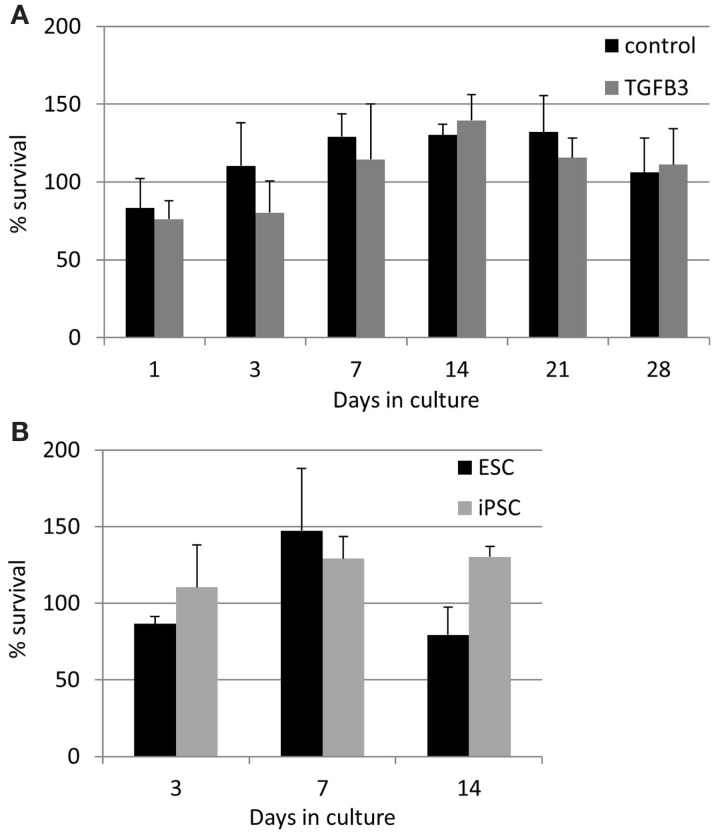
**Induced pluripotent stem cells have a high survival in the 3D constructs at all time points measured**. **(A)** The presence of TGF-β3 has no significant effect on the survival of the iPSCs. **(B)** There are no significant differences between the survival of ESCs and iPSCs up to 14 days of culture.

The lower rate of contraction by iPSCs is not due to a low survival of the cells. In contrast, they exhibit high survivals of between 75 and 140% at all time points studied (Figure 8). TGF-β3 does not have any significant effect on the survival of the iPSCs (Figure 8A), and their survival is not significantly different to that of ESCs at the time points up to 14 days which could be compared [Figure 8B; ESCs cannot be cultured in 3D beyond 14 days as they snap off the posts due to their contraction (20)].

### iPSCs Remain in Cell Clusters During 3D Culture

**Figure 9:**
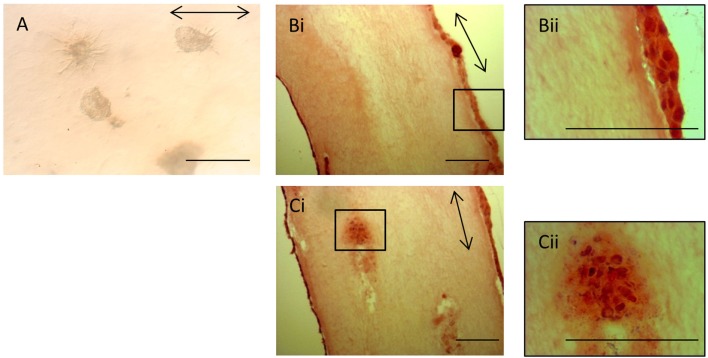
**Induced pluripotent stem cells remain in cell clusters in 3D gels**. **(A)** iPSCs in 3D constructs at 28 days remain in cell clusters. Some clusters demonstrate fibroblastic outgrowths, but these are not specifically aligned to the direction of force (arrow). **(Bi)** In contrast, H&E-stained sections of ESC 3D constructs after 14 days demonstrate that the majority of cells are at the edges of the gels and are evenly distributed. A higher magnification image is shown in **(Bii)**. **(Ci)** Occasional clusters of cells are still present in some ESC 3D constructs after 14 days. A higher magnification image is shown in **(Cii)**. Scale bar = 100 μm.

The lack of contraction of the iPSC-seeded 3D constructs meant that the constructs were too insubstantial for sectioning and so precluded analysis via histological and immunohistochemical studies. However, the iPSCs, like ESCs, were seeded as cell clusters in the 3D collagen gels. Image analysis of the iPSCs in culture demonstrated that even after 28 days in the 3D environment, all iPSCs remained in the initial clusters of cells (Figure 9A). Some of the iPSC clusters demonstrated fibroblastic outgrowths, but these occurred in all directions and did not specifically align to the direction of the intrinsic force. In contrast, H&E staining of ESC-seeded 3D constructs demonstrates that the majority of cells migrate to the outer edges of the construct and align with the direction of force (Figures 9Bi,Bii). Occasional clusters of cells remain in some gels (Figures 9Ci,Cii), but these are rare.

The iPSCs in 3D culture remain GFP negative (data not shown), suggesting that they have not re-activated the exogenous viral genes.

### iPSCs in 3D Culture Have Low Levels of Tendon Gene Expression in Comparison to ESCs

**Figure 10:**
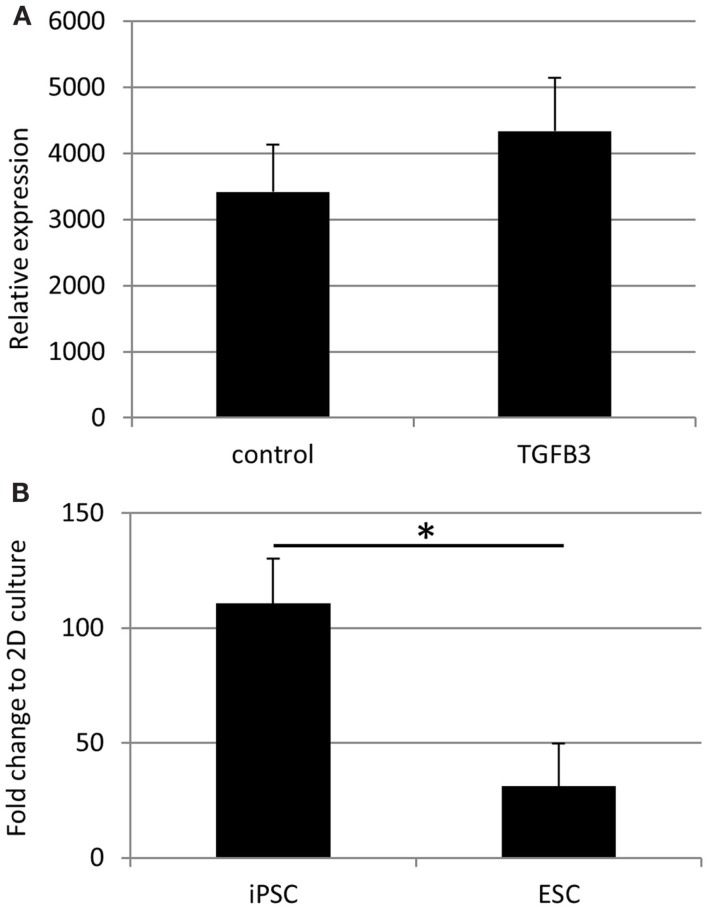
***COL1A1* gene expression by iPSCs in 3D culture**. **(A)** *COL1A1* expression in iPSCs after 28 days of 3D culture is not significantly affected by the addition of TGF-β3. **(B)** iPSCs cultured for 28 days in 3D culture without TGF-β3 show a large fold increase in their expression of *COL1A1* compared to iPSCs differentiated in 2D culture for 14 days without TGF-β3. This increase is significantly greater than that observed with ESCs cultured in 3D cultures for 14 days without TGF-β3 compared to ESCs differentiated in 2D culture for 14 days without TGF-β3.

qRT-PCR analysis has demonstrated a robust expression of tendon-associated genes in ESC collagen gels after 7 and 14 days that was much greater than the expression levels in 2D differentiation assays (20). In iPSC collagen gels cultured for 28 days in the presence or absence of TGF-β3, we could not detect robust expression of any tendon-associated genes examined with the exception of *COL1A1. COL1A1* was expressed at very high levels in iPSCs cultured in 3D gels for 28 days, and its expression was not significantly affected by the addition of TGF-β3 (Figure 10A). In comparison to iPSCs differentiated for 14 days in 2D culture in the absence of TGF-β3, 3D culture for 28 days resulted in a 110-fold increase in *COL1A1* expression. This is significantly greater than the 31-fold increase in *COL1A1* expression which occurs when ESCs are cultured in 3D for 14 days as opposed to 2D for 14 days (Figure 10B).

## Discussion

There are a number of safety issues that must be addressed prior to the clinical application of pluripotent equine stem cells. As ESCs are derived from early embryos, any future clinical application will rely on using allogeneic cells. Although we have shown promising data supporting the application of allogeneic ESCs (9, 15) further immunological assessments must be performed for specific tissue injuries where different differentiated cell types and different inflammatory environments may result in different immune responses to allogeneic cells. Although iPSCs can be derived from patients for autologous use, the time scales and expense required to do this is likely to prevent it as a treatment option. As for ESCs, there is promising data on the potential to use allogeneic equine iPSCs, but further research must be performed (24). Human iPSCs have already been taken forward to clinical trial for macular degeneration in Japan, and there is much hope that equine iPSCs will also be of clinical benefit for a range of conditions. However, to date, all equine iPSCs have been derived with integrating DNA sequences which leads to safety concerns if these sequences have the potential to become re-expressed (28) and the use of other strategies, which have successfully been employed to generate human iPSCs, using non-integrating viruses, mRNA and protein (29) need to be investigated in the horse.

Here, we compared iPSCs produced by *piggybac* transposons (21) and retroviral vectors. The retrovirally produced iPSCs generated colonies with the same morphology as transposon produced iPSCs and equine ESCs. Within a few passages, the retrovirally transduced iPSCs appeared to have silenced the expression of the exogenous viral genes, as assessed by a loss of GFP expression, which is one of the hallmarks of the pluripotent state (30). In contrast, the *piggybac* iPSCs rely on the addition of doxycycline to maintain the expression of the reprograming factors to keep the cells in the undifferentiated state (21). The retrovirally generated iPSCs express the same profile of pluripotency markers as equine ESCs (17), transposon-derived iPSCs (21) and the inner cell mass of equine embryos (17). Therefore, these markers would appear to be suitable pluripotency markers in equine cells. The retrovirally derived iPSCs also display the same ability for *in vitro* differentiation into derivatives of the three germ layers as equine ESCs (18) and transposon-derived iPSCs (21). Therefore, the method of iPSC generation used and the age of the donor fibroblasts (fetal or adult) do not appear to impact on the key pluripotency characteristics of the cells.

In order to determine the clinical potential of equine iPSCs to be used as a source of cells in tendon regeneration, we assessed their capacity for *in vitro* differentiation into functional tendon cells using the same signaling molecules and protocols which we have established for equine ESCs (19, 20). The iPSCs derived by transposons and retroviral transduction express the same tendon genes upon 2D differentiation over a 14-day time period, and this is enhanced with TGF-β3. This suggests that the method of generation and the age of the fibroblast donor (fetal or adult) do not influence the tendon differentiation capacity of the iPSCs. The iPSCs appear to be less efficient at undergoing TGF-β3-driven differentiation in 2D culture in comparison to the ESCs. The ESCs express tendon-associated genes much more quickly than the iPSCs, and the iPSCs fail to express *TNMD* at any time point studied. Interestingly, the ESCs and iPSCs do appear to express tendon-associated genes in a similar order. In both ESCs and iPSCs, tendon genes, such as *SCX* and *TNC* are first detected along with *COL1A1* suggesting that these are earlier markers of tendon differentiation than *TNMD* and *THBS4*.

We have previously shown that 3D culture is a potent driver of tendon differentiation by ESCs (20). We therefore hypothesized that, upon exposure to a combination of 3D culture and TGF-β3, the iPSCs would have a similar capacity for tendon differentiation and the generation of artificial tendons as ESCs. However, we found that iPSCs re-organize a 3D collagen gel at a significantly slower rate than ESCs. ESCs and adult tendon cells both reach their maximum contraction of the 3D collagen gels in 14 days and if left longer than this will snap off their posts (20, 31). However, even after 28 days of culture, the iPSC-containing gels did not undergo a large amount of contraction. The iPSCs survived in equivalent numbers to the ESCs, and so the lack of contraction was not simply due to a lack of survival of the cells. The iPSCs survived in constant numbers over the 28 days’ time period studied. This may reflect a constant rate of cell proliferation and death, or a partially differentiated state which does not undergo further proliferation or death. Future work using assays to determine levels of cell proliferation rather than just survival would be of benefit in understanding the fate of the iPSCs in 3D culture. As we previously observed for ESCs (20), the survival of the iPSCs was not significantly affected by the presence of TGF-β3. The iPSCs remained in cell clusters as they had been seeded and did not migrate out throughout the gel like the ESCs. We were unable to detect robust expression of any of the tendon-associated genes which they expressed in 2D cultures, with the exception of *COL1A1* which was significantly upregulated in 3D culture compared to 2D culture. Although the increase in expression of *COL1A1* in 3D culture was significantly greater than that observed for ESCs, this may reflect the length of culture as iPSCs were left for 28 days, whereas ESCs were only left for 14 days in 3D culture. The iPSCs in 3D cultures remained GFP negative, suggesting that the reprograming factors had not been re-activated. However, it remains possible that exogenous GFP could remain silenced while some or all of the exogenous pluripotency factors become re-expressed, and this could hinder the differentiation of the iPSCs in 3D. Future work to determine the expression of the exogenous reprograming genes would help determine if this was the case. Together, this suggests that the iPSCs do not efficiently undergo tendon differentiation in 3D cultures, instead remaining in a partly undifferentiated state. To further identify the state of the iPSCs, global gene expression analysis would be of great benefit to look at the expression of genes involved in pluripotency and commitment to all lineages.

This study therefore highlights the disadvantages of using gene and protein expression in 2D culture alone as a marker of successful differentiation as, after 14 days in the presence of TGF-β3, the iPSCs showed a largely comparable expression profile of tendon genes to the ESCs, and yet we were unable to demonstrate the functionality of the iPSC-derived tenocytes in remodeling a collagen matrix. We have therefore shown that our 3D culture system is a useful model to test tendon cell functionality, which is an important assay in addition to gene and protein expression studies. To date, we have not established the methodology for selecting the tenocytes from the mixed population of differentiating cells which arise in 2D differentiating cultures. However, in the future it would be of value to determine if these predifferentiated iPSCs derived in 2D culture were able to function in 3D culture.

Numerous reports have demonstrated that human iPSCs have a reduced differentiation efficiency compared to ESCs (32). This may be because differentiation protocols have been established for ESCs, and the signaling requirements of iPSCs are different. It may also be related to the fact that iPSCs retain an epigenetic memory of their origin (DNA methylation and/or histone modifications) which can affect their differentiation potential, enhancing their differentiation back to the cell type from which they were derived, and inhibiting their differentiation into other lineages (33, 34). These epigenetic changes have led to detectable changes in global gene expression profiles between human ESCs and iPSCs (35–37). In order to reduce the epigenetic memory of the iPSCs, prolonged culture may be beneficial (34) and as this study used iPSCs at fairly low passage (3–3), future work to examine the differentiation capacity of iPSCs at higher passages may be warranted. Performing the work on higher passage iPSCs may also result in a lower chance that the exogenous reprograming factors become re-expressed.

The results of this study highlight that there is still a need to understand more about pluripotency in horses to determine the mechanisms which underpin differences in differentiation potential between varying sources of pluripotent stem cells and how these differences may affect their clinical utility. Future work to compare the global gene expression and methylation patterns in ESCs versus iPSCs generated by different methods, including through the use of non-integrating viruses, is much needed before iPSCs can be taken forward as a clinical therapy.

## Author Contributions

EB, OS, and AB acquired and analyzed the data. LS and DG conceived and designed the work. DG drafted the manuscript, and all authors revised it and approved the final version for publication. All authors agree to be accountable for all aspects of the work in ensuring that questions related to the accuracy or integrity of any part of the work are appropriately investigated and resolved.

## Conflict of Interest Statement

The research was conducted in the absence of any commercial or financial relationships that could be construed as a potential conflict of interest.

## Footnotes

^1^http://frodo.wi.mit.edu/primer3

^2^http://mfold.rna.albany.edu/?q=mfold/DNA-Folding-Form
