# Supplementary figures and images for: Equine Induced Pluripotent Stem Cells have a Reduced Tendon Differentiation Capacity Compared to Embryonic Stem Cells
Source: Front Vet Sci. 2015 Nov 16;2:55. doi: 10.3389/fvets.2015.00055 (PMC4672282; doi:10.3389/fvets.2015.00055)

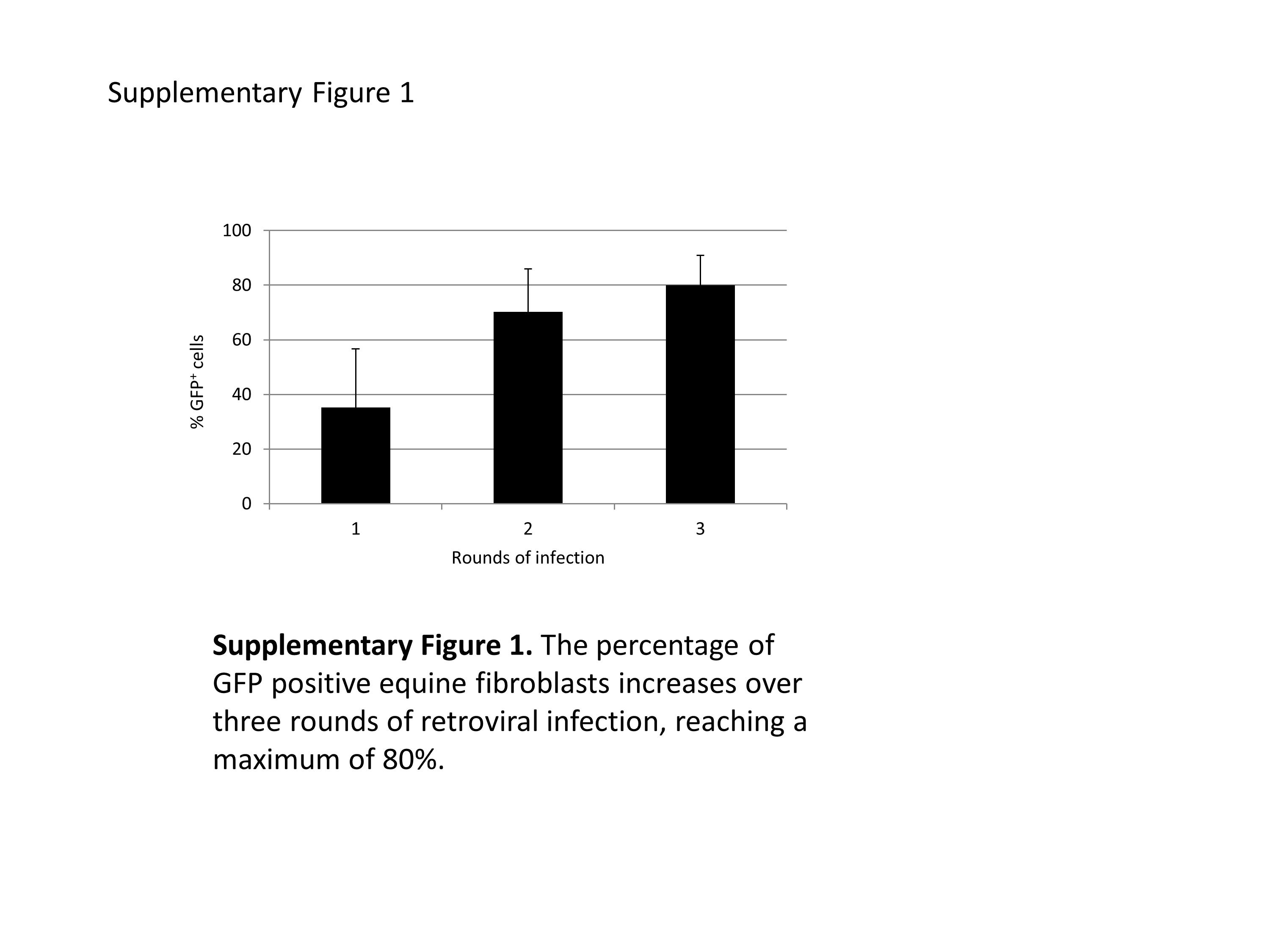

Supplement: Supplementary file 1 [file Image_1.TIF]

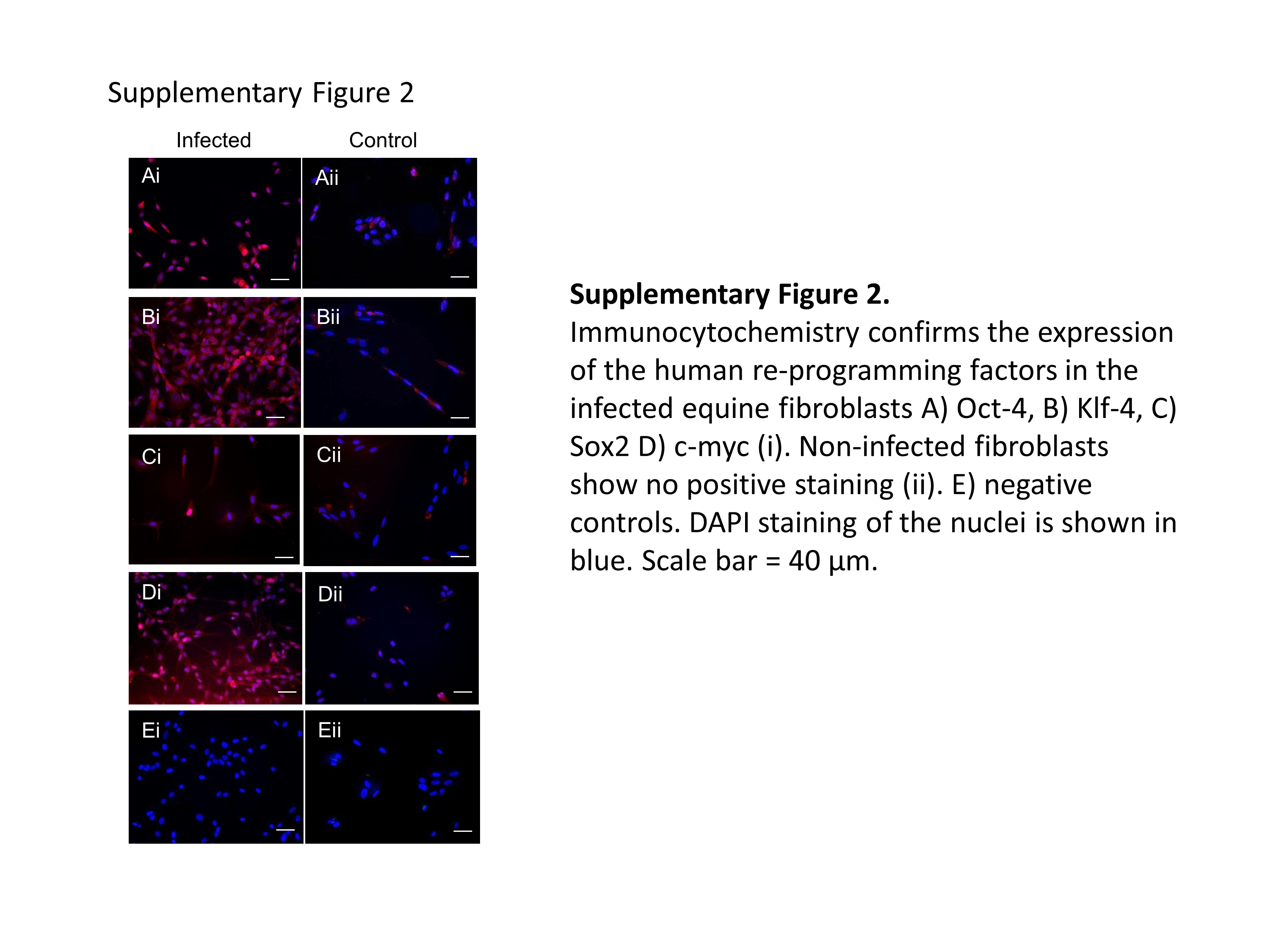

Supplement: Supplementary file 2 [file Image_2.TIF]
